# Supplementary material for: The effects of genital myiasis on the diversity of the vaginal microbiota in female Bactrian camels
Source: BMC Vet Res. 2022 Mar 5;18:87. doi: 10.1186/s12917-022-03189-5 (PMC8897907; doi:10.1186/s12917-022-03189-5)
Supplement: Supplementary file 5 — Additional file 5. [file 12917_2022_3189_MOESM5_ESM.zip › MPL201709200_16s_yy/Treat1/css/js_css/jsPDF-master.zip!/jsPDF-master/examples/html2pdf/tables.html]

Html2Pdf


# Tables

|  |  |  |  |
| --- | --- | --- | --- |
| Item | Cost | Description | Available |
| Milk | $1.00 | Hello PDF World | Out Of Stock |
| Milk | $1.00 | Hello PDF World | Out Of Stock |

|  |  |  |  |
| --- | --- | --- | --- |
| Item | Cost | Description | Available |
| Milk | $1.00 | Hello PDF World | Out Of Stock |
| Milk | $1.00 | Hello PDF World | Out Of Stock |

|  |  |  |  |
| --- | --- | --- | --- |
| Item | Cost | Description | Available |
| Milk | $1.00 | Hello PDF World | Out Of Stock |
| Milk | $1.00 | Hello PDF World | Out Of Stock |
